# Supplementary material for: Charting and probing the activity of ADARs in human development and cell-fate specification
Source: Nat Commun. 2024 Nov 13;15:9818. doi: 10.1038/s41467-024-53973-0 (PMC11561244; doi:10.1038/s41467-024-53973-0)
Supplement: Supplementary file 1 — Supplementary Information [file 41467_2024_53973_MOESM1_ESM.pdf]

SUPPLEMENTARY FIGURES

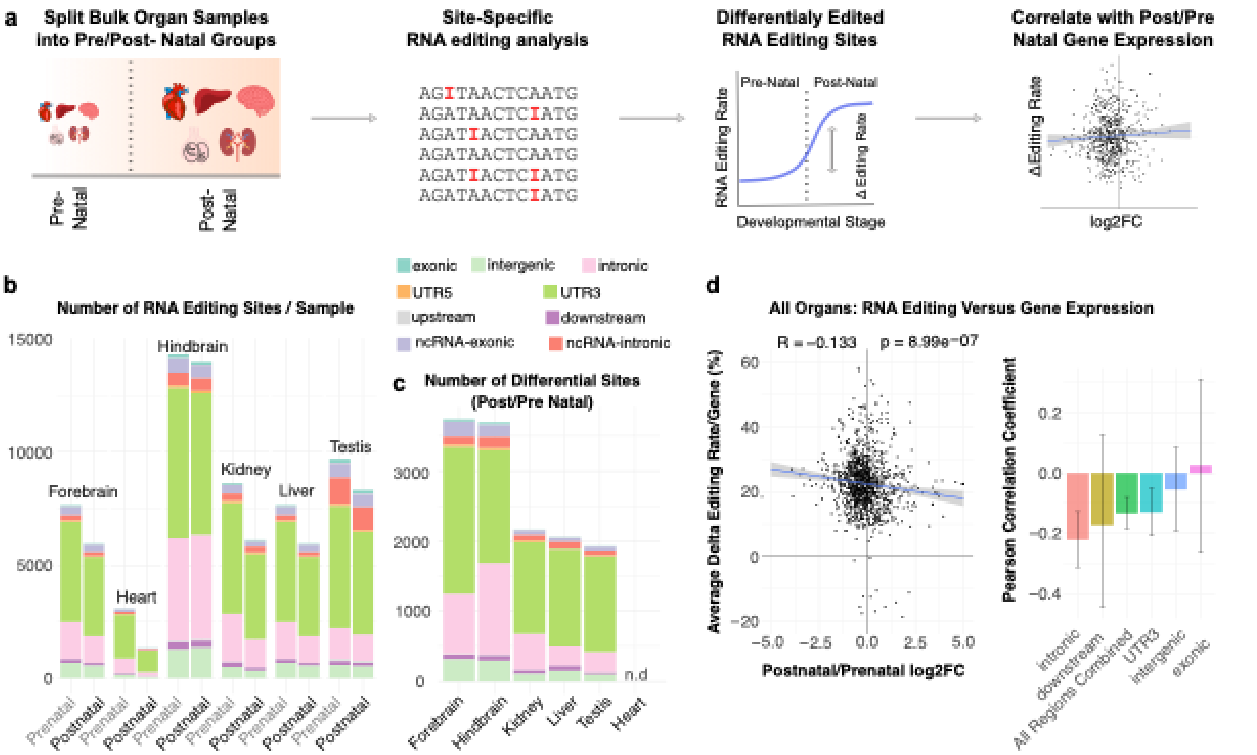

Supplementary Figure 1. Site-Specific RNA Editing Analysis in Bulk Human Organ Tissues: (A) Schematic depicting the site-specific RNA editing analysis pipeline for developmental bulk organ samples. After bulk organ samples were grouped into prenatal and postnatal groups, differential editing analysis was conducted between these two developmental groups. Finally, prenatal versus postnatal differences in RNA editing levels (delta editing rate) were correlated with corresponding gene expression level changes (log fold-change). (B) Total number of RNA editing sites discovered across all examined organs. (C) Total number of prenatal versus postnatal differentially edited RNA editing sites discovered across all examined organs (n.d. = none detected). (D) Prenatal versus postnatal differences in RNA editing levels (delta editing rate/gene) were correlated with corresponding gene expression level changes (log fold-change/gene) for all genes captured across all organs. Delta editing rates per gene were averaged across all delta values for each site per unique gene. Correlation was measured using a Pearson's Correlation Coefficient and 95% confidence intervals are plotted for each genetic region.

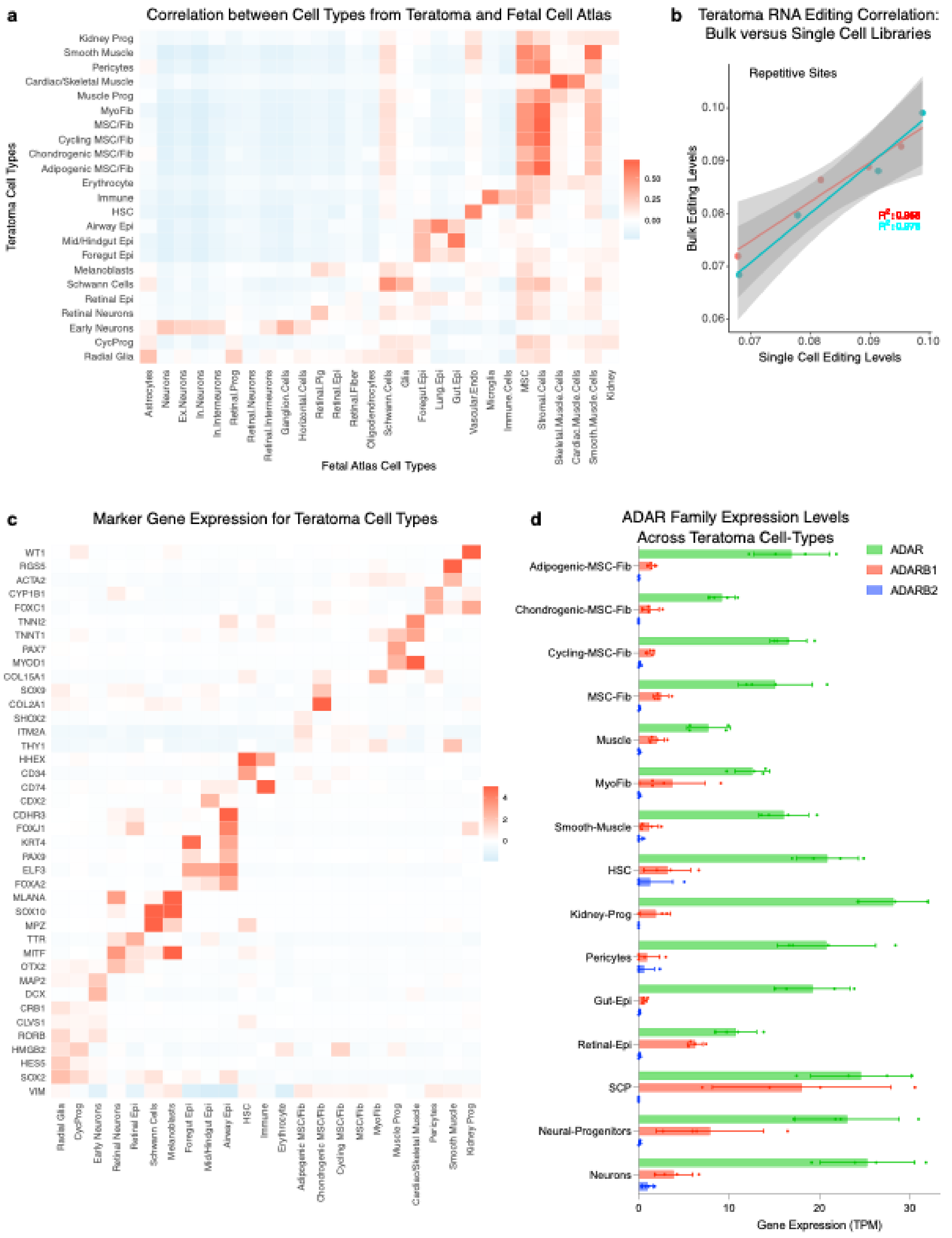

Supplementary Figure 2. Single Cell RNA Editing Analysis in WT Teratomas: (A) Transcriptomic correlation between teratoma cell-types and cells from the Human Fetal

Cell Atlas database. (B) Correlation between bulk teratoma and single cell teratoma RNA sequencing editing levels across repetitive editing sites. (C) Marker gene expression for all teratoma cell-types. (D) *ADAR*, *ADARB1*, and *ADARB2* expression levels for all teratoma cell-types. Error bars represent the standard error of the mean.

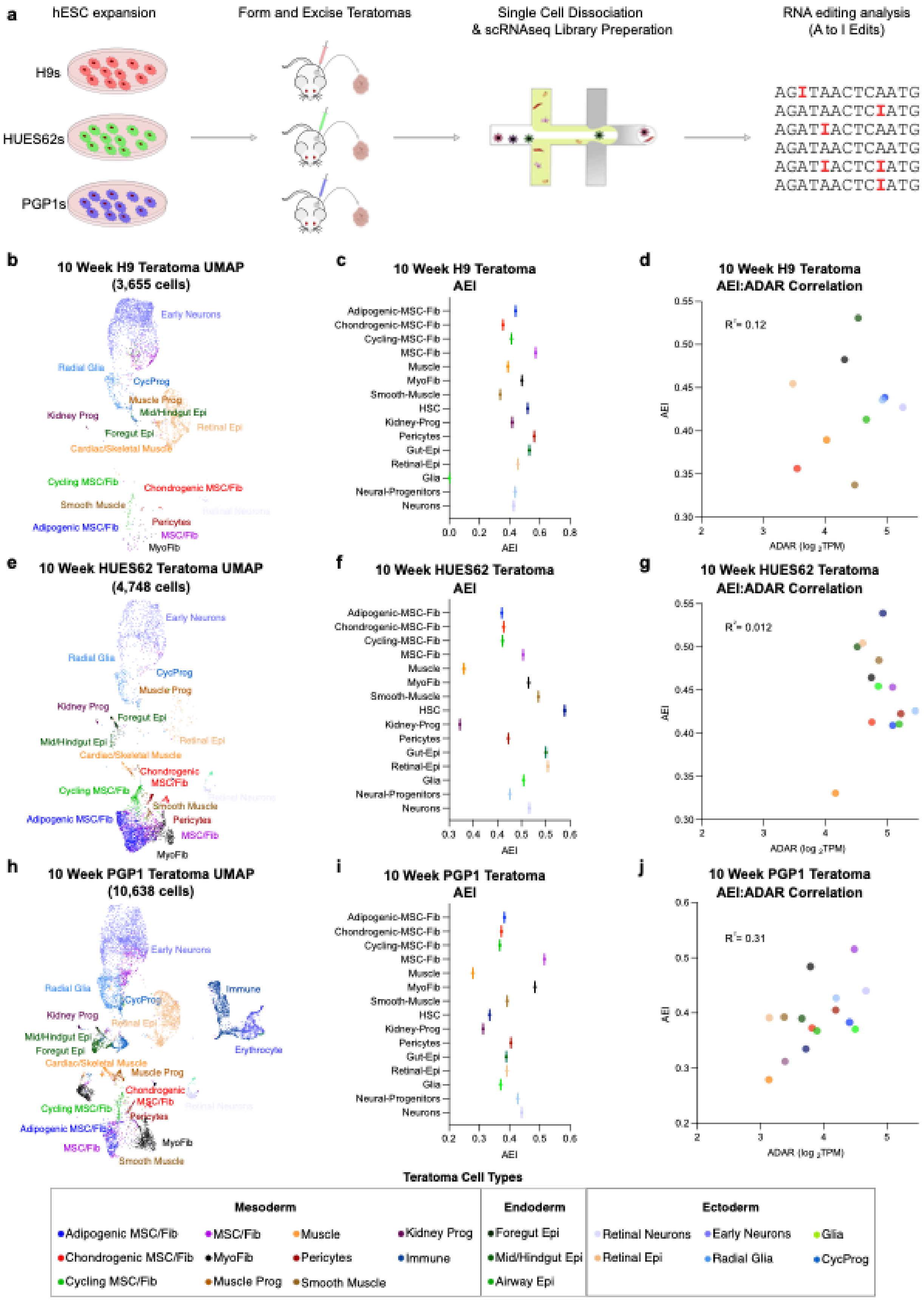
a
hESC expansion
Form and Excise Teratomas
Single Cell Dissociation & scRNAseq Library Preperation
RNA editing analysis (A to I Edits)
H9s
HUES62s
PGP1s
b
10 Week H9 Teratoma UMAP (3,655 cells)
c
10 Week H9 Teratoma AEI
d
10 Week H9 Teratoma AEI:ADAR Correlation
e
10 Week HUES62 Teratoma UMAP (4,748 cells)
f
10 Week HUES62 Teratoma AEI
g
10 Week HUES62 Teratoma AEI:ADAR Correlation
h
10 Week PGP1 Teratoma UMAP (10,638 cells)
i
10 Week PGP1 Teratoma AEI
j
10 Week PGP1 Teratoma AEI:ADAR Correlation
Teratoma Cell Types
Mesoderm
Endoderm
Ectoderm
Adipogenic MSC/Fib
Chondrogenic MSC/Fib
Cycling MSC/Fib
MSC/Fib
MyoFib
Muscle Prog
Muscle
Pericytes
Smooth Muscle
Kidney Prog
Immune
Foregut Epi
Mid/Hindgut Epi
Airway Epi
Retinal Neurons
Retinal Epi
Early Neurons
Radial Glia
Glia
CycProg

Supplementary Figure 3. RNA editing and ADAR expression analysis in alternative PSC lines: (A) Workflow schematic for generating teratomas from various PSC lines and conducting downstream single-cell RNA editing analysis across alternative lines. (B) UMAP plot from H9 teratoma processed through the single cell RNA sequencing pipeline. (C) AEI values for H9 teratoma cell-types, with each data point calculated as a pseudo-bulk value from each H9 teratoma cell-type (n=1 teratoma). (D) Correlation between AEI value to *ADAR* expression for all H9 teratoma cell-types. (E) UMAP plot from HUES62 teratoma processed through the single cell RNA sequencing pipeline. (F) AEI values for HUES62 teratoma cell-types, with each data point calculated as a pseudo-bulk value from each HUES62 teratoma cell-type (n=1 teratoma). (G) Correlation between AEI value to *ADAR* expression for all HUES62 teratoma cell-types. (H) UMAP plot from PGP1 teratoma processed through the single cell RNA sequencing pipeline. (I) AEI values for PGP1 teratoma cell-types, with each data point calculated as a pseudo-bulk value from each PGP1 teratoma cell-type (n=1 teratoma). (J) Correlation between AEI value to *ADAR* expression for all PGP1 teratoma cell-types.

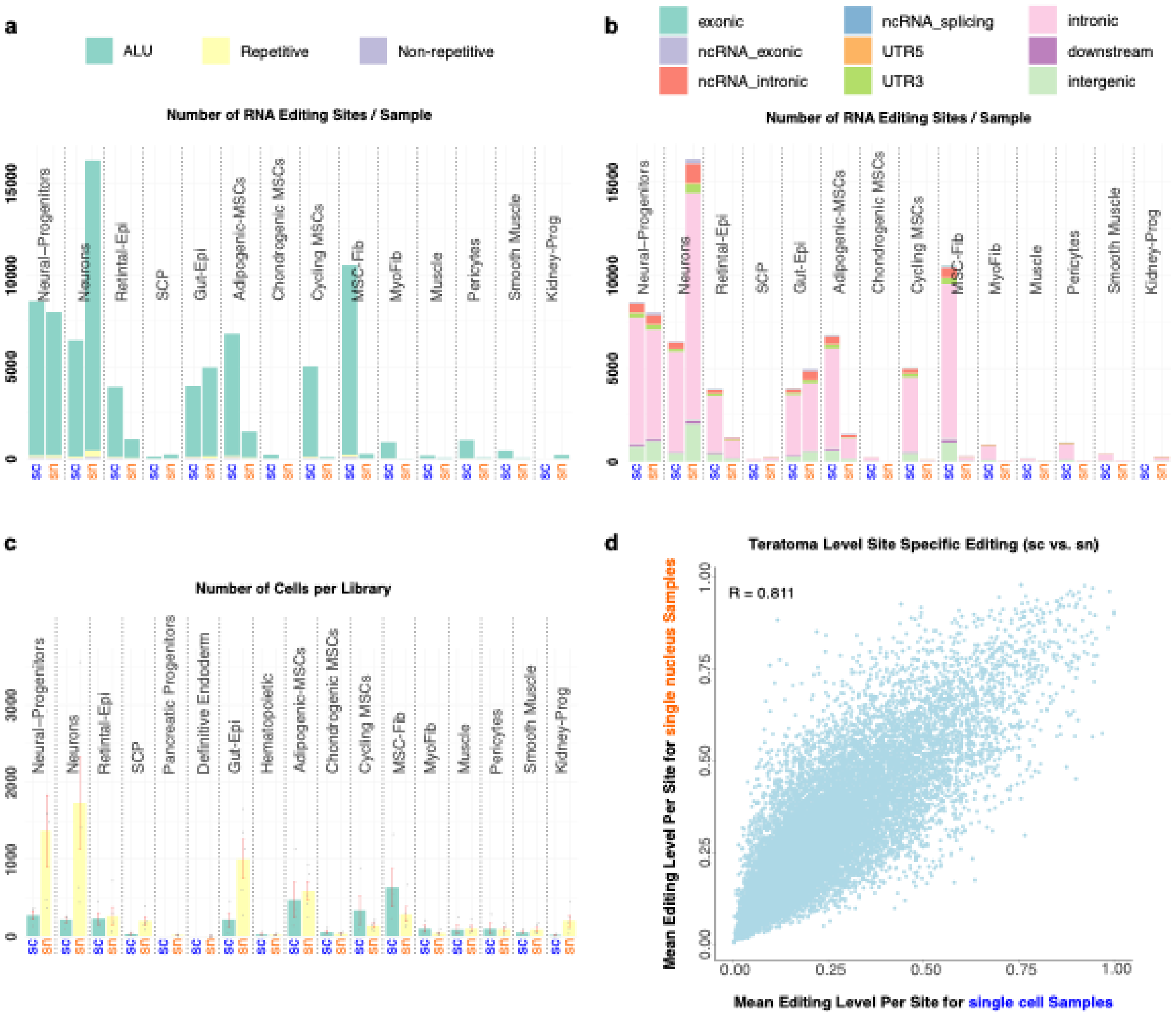

Supplementary Figure 4. Comparison between snRNAseq and scRNAseq library preparation modalities for RNA editing analysis: (A-B) The number of captured RNA editing sites between single cell and single nucleus library preparation modalities, across teratoma cell-types. Sites are reported as either being from repetitive DNA sequences (A) or their annotated genetic region (B) Error bars represent the standard error of the mean. (C) The number of captured cells between single cell and single nucleus library preparation modalities, across teratoma cell-types. Error bars represent the standard error of the mean. (D) The correlation in RNA editing rates between matching sites captured in single cell and single nucleus RNA sequencing libraries.

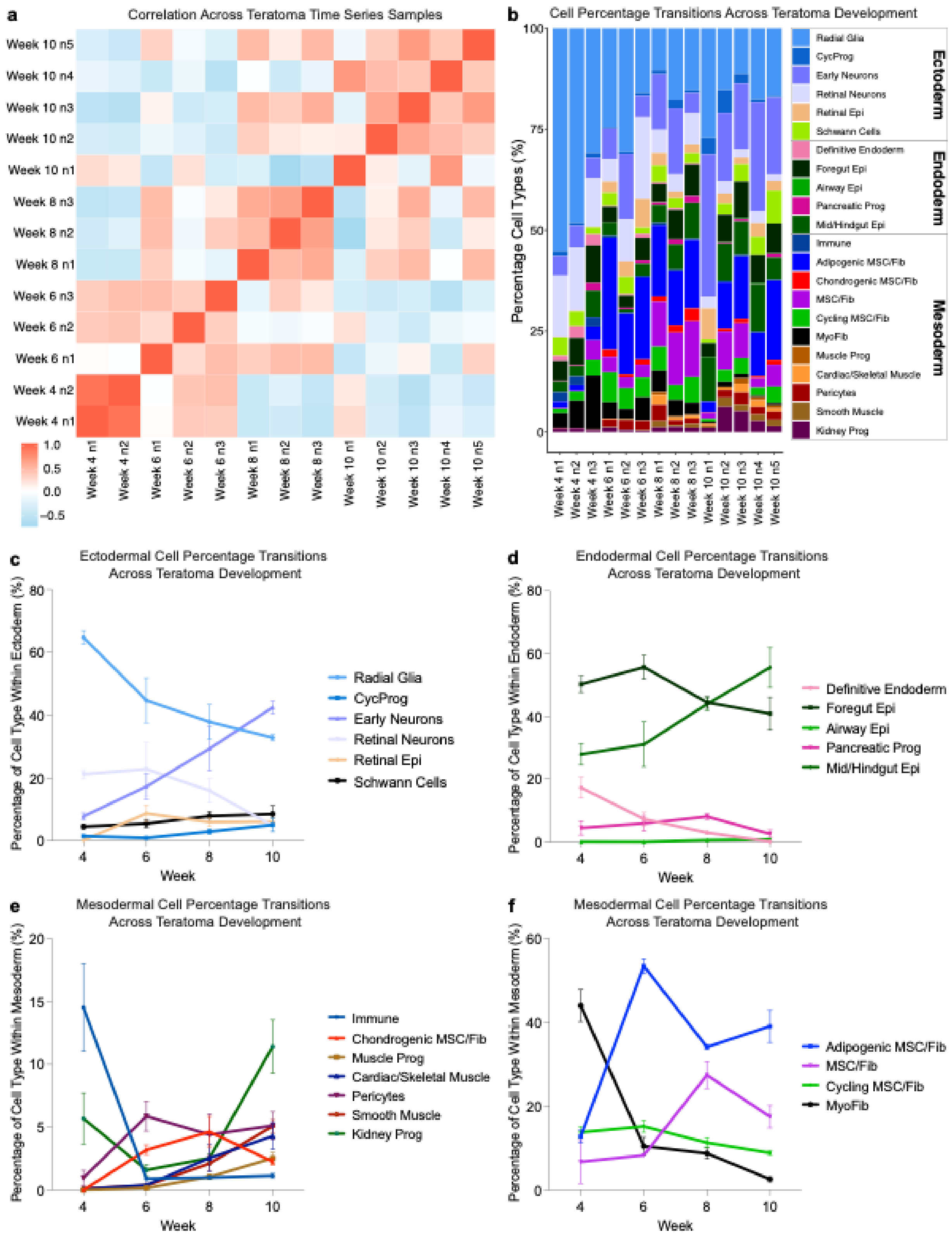

Supplementary Figure 5. Percent cell type variation across teratoma development: (A) Transcriptomic correlation across teratoma time series samples, depicting higher

levels in comparisons correlation from teratomas samples of the same developmental time point. (B) Percentage of all teratoma cell-types across development, for each teratoma sample. (C) Ectodermal cell-types proportions across teratoma development. (D) Endodermal cell-types proportions across teratoma development. (E) Sub-set of mesodermal cell-types proportions across teratoma development. (F) Sub-set of remaining mesodermal cell-types proportions across teratoma development. Cell-type percentages are normalized within each germ-layer. (C-F) Error bars represent the standard error of the mean.

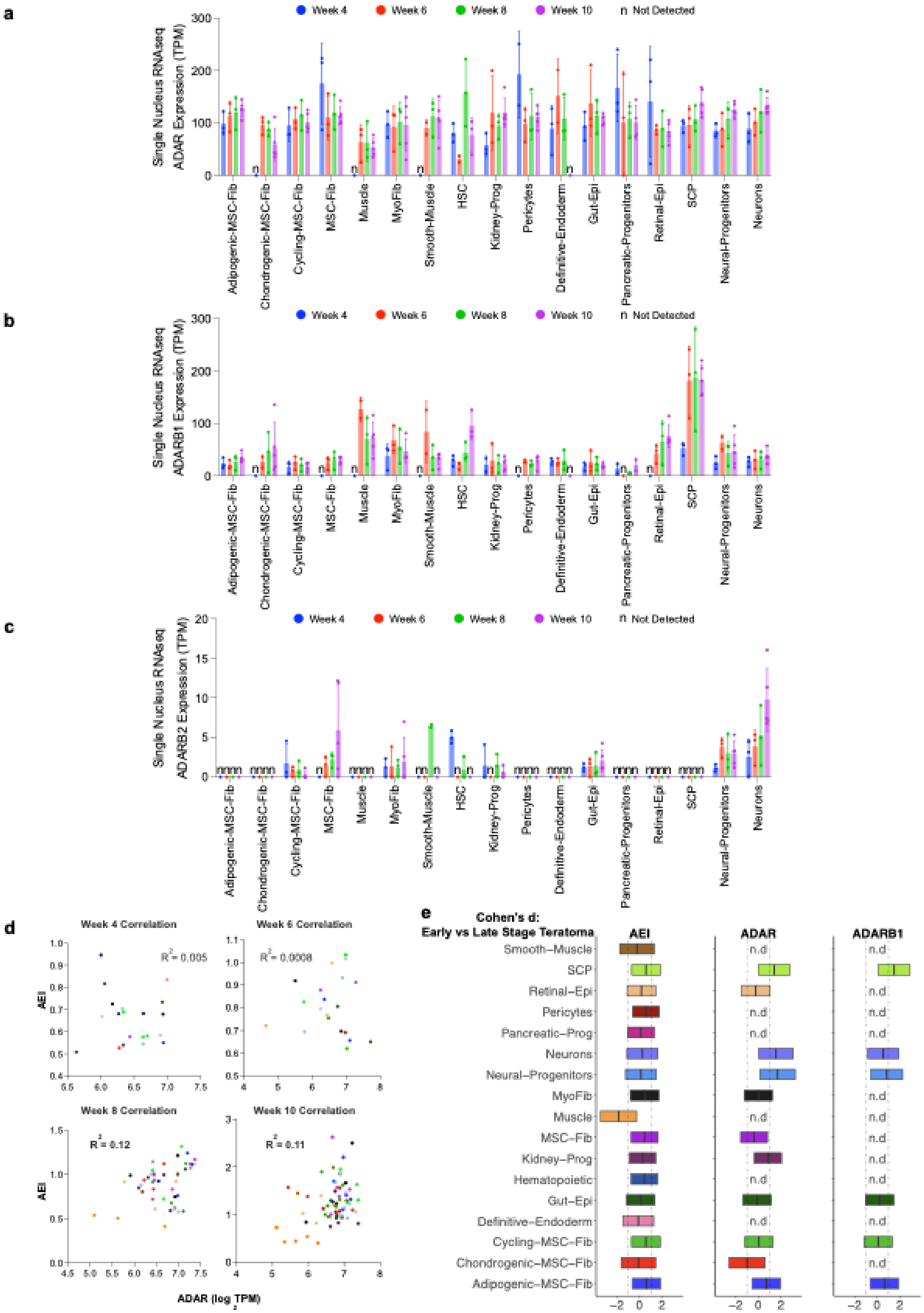
a
Week 4
Week 6
Week 8
Week 10
n Not Detected
Single Nucleus RNAseq ADAR Expression (TPM)
Adipogenic-MSC-Fib
Chondrogenic-MSC-Fib
Cycling-MSC-Fib
MSC-Fib
Muscle
MyoFib
Smooth-Muscle
HSC
Kidney-Prog
Pericytes
Definitive-Endoderm
Gut-Epi
Pancreatic-Progenitors
Retinal-Epi
SCP
Neural-Progenitors
Neurons
b
Single Nucleus RNAseq ADARB1 Expression (TPM)
c
Single Nucleus RNAseq ADARB2 Expression (TPM)
d
Week 4 Correlation
R^2 = 0.005
Week 6 Correlation
R^2 = 0.0008
Week 8 Correlation
R^2 = 0.12
Week 10 Correlation
R^2 = 0.11
AEI
ADAR ($\log_2$ TPM)
e
Cohen's d:
Early vs Late Stage Teratoma
AEI
ADAR
ADARB1
Smooth–Muscle
SCP
Retinal–Epi
Pericytes
Pancreatic–Prog
Neurons
Neural–Progenitors
MyoFib
Muscle
MSC–Fib
Kidney–Prog
Hematopoietic
Gut–Epi
Definitive–Endoderm
Cycling–MSC–Fib
Chondrogenic–MSC–Fib
Adipogenic–MSC–Fib
n.d

Supplementary Figure 6. ADAR enzyme family expression in teratoma cell types across time: (A) *ADAR* expression values for all teratoma cell-types, across teratoma developmental time-points. (B) *ADARB1* expression values for all teratoma cell-types, across teratoma developmental time-points. (C) *ADARB2* expression values for all teratoma cell-types, across teratoma developmental time-points. (A-C) Error bars represent the standard error of the mean. (D) AEI to *ADAR* expression correlation for all teratoma cell-types, across teratoma developmental time-points. (E) Cohen's d comparison between Early Teratoma (weeks 4+6) samples versus Late Teratoma (weeks 8+10) samples for AEI, *ADAR*, *ADARB1*, and *ADARB2*, across all teratoma cell-types (not detected = n.d). Center of the bar represents the mean and the box represents the 95% confidence interval.

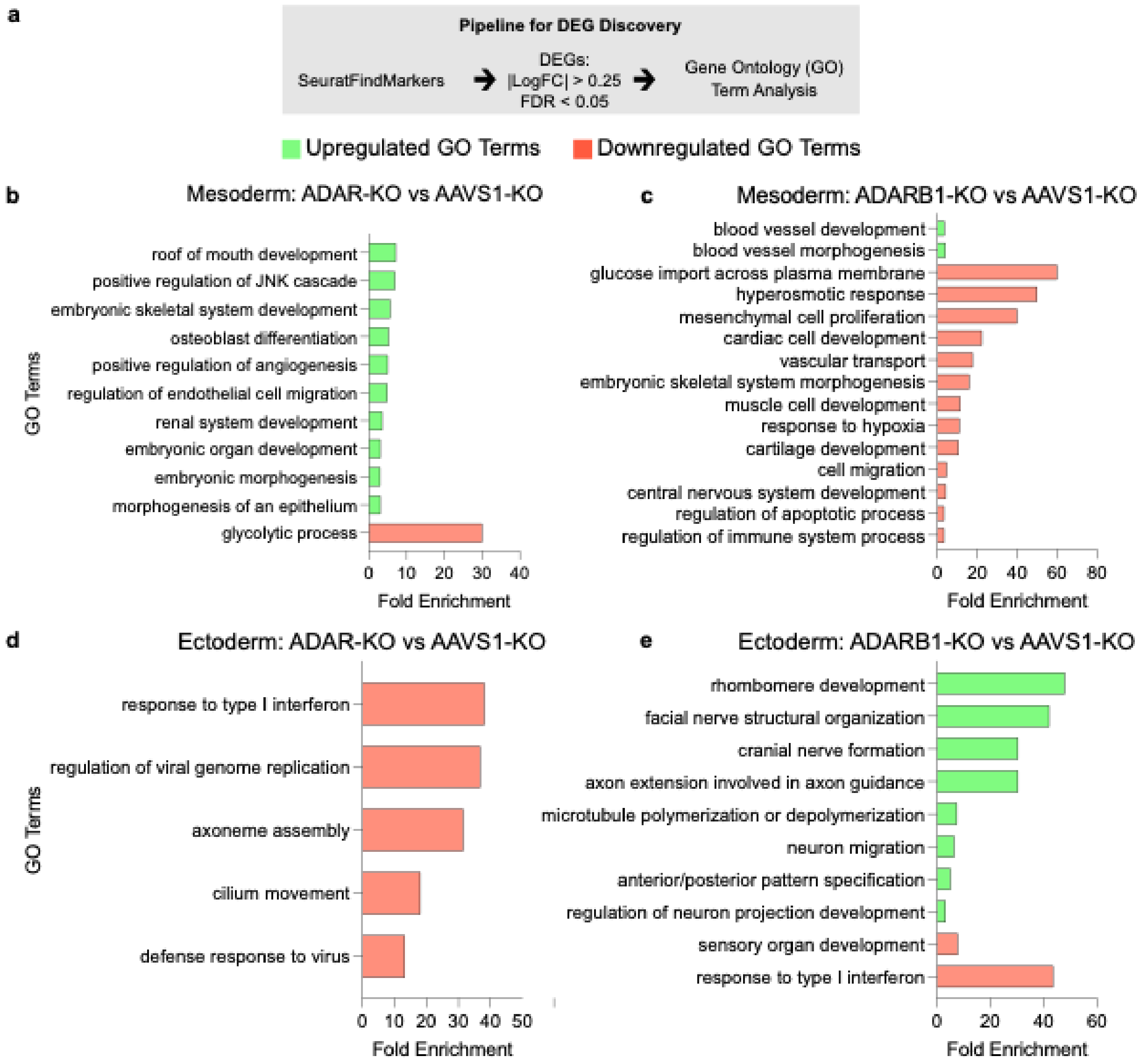

Supplementary Figure 7. Gene Ontology Discovery Pipeline for ADAR-KO Teratomas: (A) Inflammatory and immune gene panel expression for germ-layer *ADAR*-KO samples, normalized to *AAVS1*-KO expression. Values represented as Log2(Fold Change). (A) Computational pipeline for DEG discovery. (B) Top GO terms identified from Mesoderm *ADAR*-KO versus *AAVS1*-KO DEGs discovery pipeline. (C) Top GO terms identified from Mesoderm *ADARB1*-KO versus *AAVS1*-KO DEGs discovery pipeline. (D) Top GO terms identified from Ectoderm *ADAR*-KO versus *AAVS1*-KO DEGs discovery pipeline. (E) Top GO terms identified from Ectoderm *ADARB1*-KO versus *AAVS1*-KO DEGs discovery pipeline.

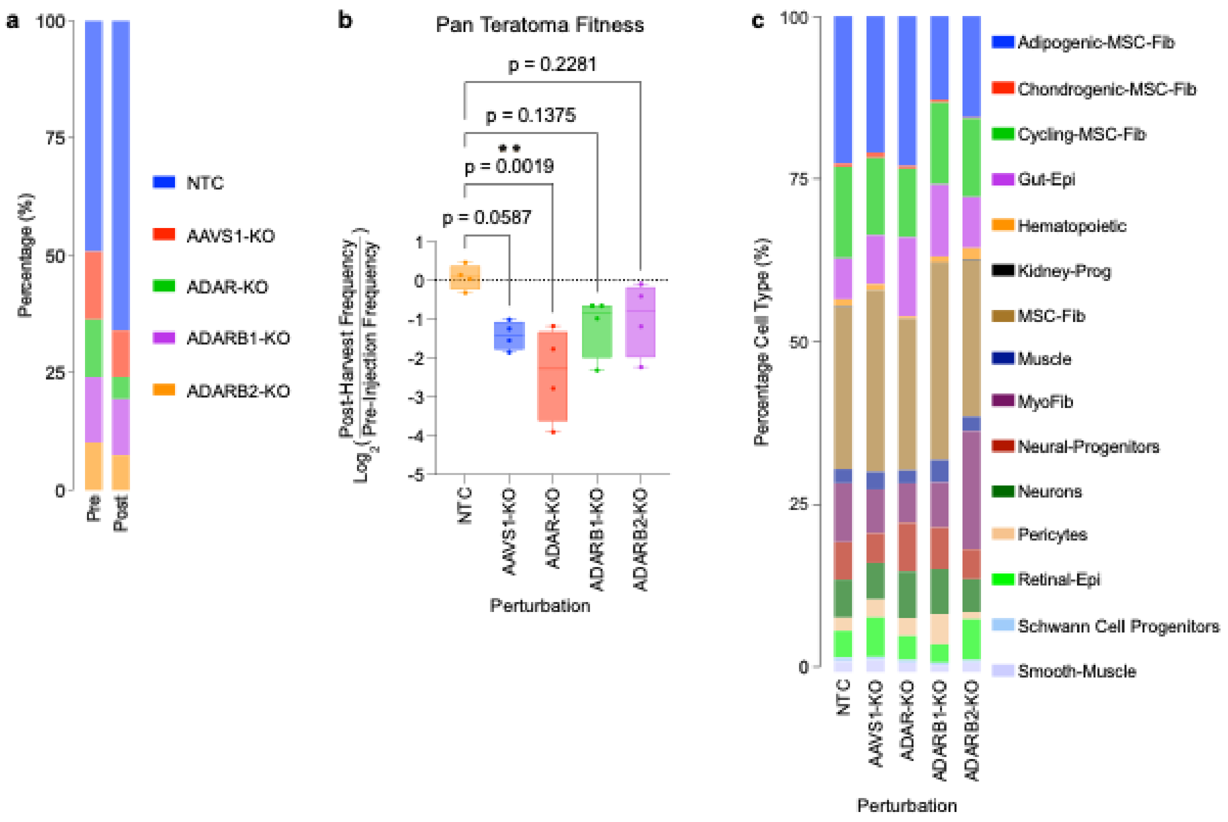

Supplementary Figure 8. Pan-Teratoma View of ADAR-KO Impact on Cell Fitness: (A) Pan-teratoma percentage for each perturbation in the pre-injection population and post-teratoma harvest. (B) Pan-teratoma fitness analysis across perturbation conditions in ADAR-KO teratomas. (n=4 teratoma biological replicates, error bars represent standard error of the mean). (*p ≤ 0.05, **p ≤ 0.01, ***p ≤ 0.001, and ****p ≤ 0.0001; ns, not significant.) (C) Percentage of all teratoma cell-types across all perturbations.

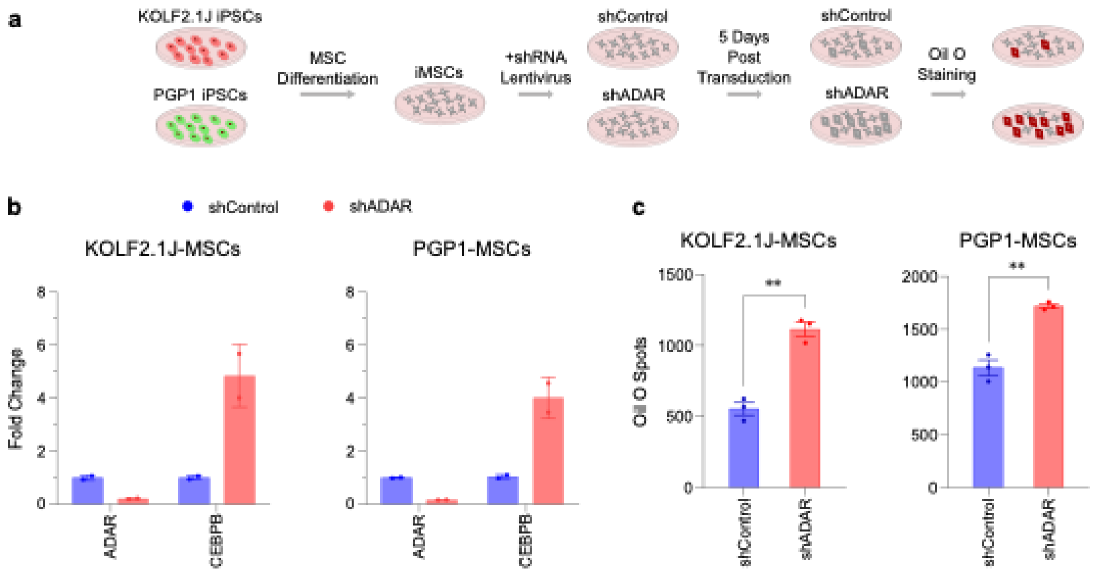

Supplementary Figure 9. Enhanced Adipogenesis in iPSC-MSCs following *ADAR*-KO: (A) Schematic depicting experimental workflow for transducing iPSC derived MSCs with lentivirus encoding shRNA targeting *ADAR* gene expression, and subsequently checking for its effect on adipogenesis (B) *ADAR* and adipogenic transcription factor *CEBPB* transcript quantification in iPSC derived MSCs 5 days post shRNA transduction. (C) Quantification of oil o red staining spots in iPSC derived MSCs cultures 5 days post transduction. (n=3, error bars represent standard error of the mean); (*p ≤ 0.05, **p ≤ 0.01, ***p ≤ 0.001, and ****p ≤ 0.0001; ns, not significant).

Supplementary Table 1 : ADAR-KO gRNA amplification primers

ADAR1_1_F:	TATATATCTTGTGGAAAGGACGAAACACCGCGTTCTCCCCAATCAAGACA
ADAR1_2_F:	TATATATCTTGTGGAAAGGACGAAACACCGTTCTTGTAGGGTGAACACCG
ADAR1_3_F:	TATATATCTTGTGGAAAGGACGAAACACCGACTCCAAAAGGCCACCCACA
ADAR1_4_F:	TATATATCTTGTGGAAAGGACGAAACACCGTCTACAGTCATGGCTTGCCA
ADARB1_1_F:	TATATATCTTGTGGAAAGGACGAAACACCGGGTGAATACATGAGTGATCG
ADARB1_2_F:	TATATATCTTGTGGAAAGGACGAAACACCGTCAGGTCACCAAACTTACCC
ADARB1_3_F:	TATATATCTTGTGGAAAGGACGAAACACCGTTGGAGCCCACGTAAAAGGG
ADARB1_4_F:	TATATATCTTGTGGAAAGGACGAAACACCGAGTACCGCCTGAAGAAAAGG
ADARB2_1_F:	TATATATCTTGTGGAAAGGACGAAACACCGGGCGTTGGGGAACTGCACGA
ADARB2_2_F:	TATATATCTTGTGGAAAGGACGAAACACCGGTGGACGGCAGGACGTTCGA
ADARB2_3_F:	TATATATCTTGTGGAAAGGACGAAACACCGTCCTGGCATCACAAACACGG
ADARB2_4_F:	TATATATCTTGTGGAAAGGACGAAACACCGGAAGAAGGCCAAGATGCGCG
ADAR1_1_R:	ATTTTAACTTGCTATTTCTAGCTCTAAAACTGTCTTGATTGGGGAGAACG
ADAR1_2_R:	ATTTTAACTTGCTATTTCTAGCTCTAAAACCGGTGTTCACCCTACAAGAA
ADAR1_3_R:	ATTTTAACTTGCTATTTCTAGCTCTAAAACTGTGGGTGGCCTTTTGGAGT
ADAR1_4_R:	ATTTTAACTTGCTATTTCTAGCTCTAAAACTGGCAAGCCATGACTGTAGA
ADARB1_1_R:	ATTTTAACTTGCTATTTCTAGCTCTAAAACCGATCACTCATGTATTCACC
ADARB1_2_R:	ATTTTAACTTGCTATTTCTAGCTCTAAAACGGGTAAGTTTGGTGACCTGA
ADARB1_3_R:	ATTTTAACTTGCTATTTCTAGCTCTAAAACCCCTTTTACGTGGGCTCCAA
ADARB1_4_R:	ATTTTAACTTGCTATTTCTAGCTCTAAAACCCTTTTCTTCAGGCGGTACT
ADARB2_1_R:	ATTTTAACTTGCTATTTCTAGCTCTAAAACTCGTGCAGTTCCCCAACGCC
ADARB2_2_R:	ATTTTAACTTGCTATTTCTAGCTCTAAAACTCGAACGTCCTGCCGTCCAC
ADARB2_3_R:	ATTTTAACTTGCTATTTCTAGCTCTAAAACCCGTGTTTGTGATGCCAGGA
ADARB2_4_R:	ATTTTAACTTGCTATTTCTAGCTCTAAAACCGCGCATCTTGGCCTTCTTC

SUPPLEMENTARY METHODS

Cell Culture

H1, H9, HUES62, KOLF2.1Js, and PGP1 PSC cell lines were maintained under feeder-free conditions in mTeSR1 medium (Stem Cell Technologies 85850). Prior to passaging, tissue-culture plates were coated with growth factor-reduced Matrigel (Corning 356230) diluted in DMEM/F-12 medium (Thermo Fisher Scientific 11320033) and incubated for 30 minutes at 37 ºC, 5% CO2. Cells were dissociated and passaged using the dissociation reagent Versene (Thermo Fisher Scientific 15040066).

Primary human MSCs (Lonza PT-2501) were cultured in MSCGM (Lonza PT-3001) and passaged with Trypsin-EDTA 0.05% (Thermo Fisher Scientific 25300054). To induce adipogenic fate, MSC media was replaced with Stempro Adipogenic Induction Media (Thermo Fisher Scientific A1007001) for 8 days

PGP1-Cas9 Cell Line Generation

The PGP1-Cas9 line was generated and comprehensively characterized in a previous publication [1].

ADAR Targeting Guide Library Generation

To select the ADAR knockout sgRNA guides sequences, guides that targeted the *ADAR*, *ADARB1* and *ADARB2* genes were taken from the Brunello knockout library [2] and from the Achilles knockout library (https://depmap.org/portal/download/all/), giving a total of eight guides per gene. The guides were then compared using the CRISPR-Cas9 guide RNA design checker from IDT (https://www.idtdna.com/site/order/designtool/index/CRISPR_SEQUENCE), and four guides per gene were selected that had maximum on target editing, and minimal off target editing predictions. These guides were subsequently cloned into CROPseq-Guide-Puro backbone (Addgene: #86708). The CROPseq-Guide-Puro backbone was digested with BsmBI (NEB #R0580) for 1 hour. gRNA primers were amplified using KAPA HiFi HotStart Ready Mix (Roche KK2601) using the corresponding Forward (F) and Reverse (R) primers at the following conditions: 95ºC for 3min; 12 cycles of: 98ºC for 20sec, 65ºC for 15sec, 72ºC for 15sec; 72ºC for 1min. gRNA primers for creating ADAR gRNA amplicons were ordered from Integrated DNA Technologies, and are included as a supplementary table.

The Gibson assembly reactions were set up as follows: 100 ng digested backbone, 3:10 molar ratio of insert, 2X Gibson assembly master mix (New England Biolabs), H20 up to

20 µl. After incubation at 50 °C for 1 h, the product was transformed into One Shot Stbl3 chemically competent Escherichia coli (Invitrogen). A fraction (150 µL) of cultures was spread on carbenicillin (50 µg/ml) LB plates and incubated overnight at 37 ºC. Individual colonies were picked, introduced into 5 ml of carbenicillin (50 µg/ml) LB medium and incubated overnight in a shaker at 37 ºC. The plasmid DNA was then extracted with a QIAprep Spin Miniprep Kit (Qiagen), and Sanger sequenced to verify correct assembly of the vector. Following verification of the vector, larger amounts of plasmid were obtained by seeding 150 µl of bacterial stock into 150 ml of LB medium containing carbenicillin (50 µg/ml) and incubating overnight in a shaker at 37 ºC for 16-18 h. The plasmid DNA was then extracted using a Plasmid Maxi Kit (Qiagen).

ADAR-KO Library Lentiviral Production

HEK 293T cells were maintained in high glucose DMEM supplemented with 10% fetal bovine serum (FBS). Cells were seeded in a 15 cm plate 1 day prior to transfection, such that they were 60–70% confluent at the time of transfection. For each plate, 36 µl of Lipofectamine 2000 (Thermo Fisher Scientific 11668027) was added to 2 mL of Opti-MEM (Thermo Fisher Scientific 31985062). Separately 3 µg of pMD2.G (Addgene #12259), 12 µg of pCMV delta R8.2 (Addgene #12263) and 9 µg of the pooled gRNA vector library was added to 2 mL of Opti-MEM. After 5 minutes of incubation at room temperature, the Lipofectamine 2000 and DNA solutions were mixed and incubated at room temperature for 30 minutes. During the incubation period, medium in each plate was replaced with 20 ml of fresh, pre-warmed medium per well. After the incubation period, the mixture was added dropwise to each plate of HEK 293T cells. Supernatant containing the viral particles was harvested after 48 and 72 hours, filtered with 0.45 µm filters (Steriflip, Millipore), and further concentrated using Amicon Ultra-15 centrifugal ultrafilters with a 100,000 NMWL cutoff (Millipore) down to a final volume of 600–800 µl, for each 15 cm plate. Finally, the concentrated supernatant was divided into 200 µl aliquots and frozen at −80°C. Two 15 cm plates worth of lentivirus was adequate.

ADAR-KO Library Lentiviral Transduction

For viral transduction, on day −1, PGP1-Cas9 cells were dissociated to a single cell suspension using Accutase and seeded into Matrigel-coated 6-well plates at a density of 300,000 cells per well in mTeSR containing ROCK inhibitor, Y27632 (10 µM, Tocris 1254). The next day, day 0, cells were approximately 20% confluent. Medium containing Y27632 was replaced with mTeSR1 within 16 hours after plating and cells were allowed to recover for at least 8 hours prior to addition of virus.

Recovered cells were then transduced with lentivirus added to fresh mTeSR containing polybrene (5 μg/ml, Millipore TR1003G). On day 1, medium was replaced with fresh mTeSR1. Puromycin (Thermo Fisher Scientific J67236-XF) selection was started from day 2 onward at a selection dose of 1 μg/ml. Medium containing puromycin was replaced daily, and cells were harvested for teratoma formation after 5 days of selection.

Teratoma Formation

A subcutaneous injection of 6–8 million PSCs in a slurry of growth factor reduced Matrigel (Corning 356230) and mTeSR medium (Stem Cell Technologies 85850) (1:1) was made in the right flank of anesthetized, 6–8 week old, male Rag2−/−;γc−/− immunodeficient mice. Weekly monitoring of teratoma growth was made by quantifying approximate elliptical area (mm2) with the use of calipers measuring outward width and height. When a tumor size of at least 10 mm in any direction, daily monitoring is conducted. Teratomas are grown for up to 10 weeks prior to harvesting.

All animal procedures were performed in accordance with protocol number S16003 approved by the Institutional Animal Care and Use Committee of the University of California, San Diego.

Teratoma Single Cell Dissociation

Mice were euthanized by slow release of CO_2 followed by secondary means via cervical dislocation. Tumor area was shaved, sprayed with 70% ethanol, and then extracted via surgical excision using scissors and forceps. Tumor was rinsed with PBS, weighed, and photographed. Tumor was then cut into small 1 inch pieces in a semi-random fashion and frozen in OCT for sectioning and H&E staining courtesy of the Moore's Cancer Center Histology Core. Remaining tumor was cut into small pieces 1–2 mm in diameter and subjected to standard GentleMACS (Miltenyi) protocols: Human Tumor Dissociation Kit (medium tumor settings) and Red Blood Cell Lysis Kit. For single cell RNA-seq, samples were also processed with the Dead Cell Removal Kit (Miltenyi). Single cells were then resuspended in PBS + 0.04% BSA for processing on the 10X Genomics Chromium [3] platform and downstream sequencing on an Illumina NovaSeq platform. For bulk processing, after red blood cell removal, cells were divided into multiple tubes and pelleted for 5 minutes at 300g. Supernatant was then removed and cells were either directly frozen at −80°C or resuspended in RNALater (Thermo Fisher Scientific AM7021), incubated overnight at 4°C, RNALater removed and then frozen at −80°C.

Single Cell Sequencing Library Generation

Cells were passed through a 40 μm filter, spun down at 300 rcf for 5 minutes, and resuspended in 0.04% BSA solution. Cells were then loaded into the Chromium Chip B (10x Genomics) and single cell libraries were made using Chromium Single Cell 3' Reagent Kits v3 workflow (10x Genomics). Fastq files were aligned to a hg38 reference and expression matrices were generated using the count command in cellranger v3.0.1 (10X Genomics).

ADAR-KO gRNA amplification

gRNA barcodes were amplified from ADAR-KO teratoma cDNA generated by the 10x single cell platform, and gDNA from the same cells harvested pre-teratoma injection. Amplicons were prepared for deep sequencing through a two-step PCR process.

For amplification of barcodes from cDNA, the first step was performed as four separate 50 µl reactions for each sample. 2.5 µl of the cDNA was input per reaction with Kapa Hifi Hotstart ReadyMix (Kapa Biosystems KK2602). The PCR primers used were, CROPseq_sgRNA_barcodeAmp_Fprimer: GACTGGAGTTCAGACGTGTGCTCTTCCGATCTCTTGTGGAAAGGACGAAACAC and NEBNext Universal PCR Primer for Illumina (New England Biolabs). The thermocycling parameters were 95°C for 3 min; 20–26 cycles of (98°C for 20 s; 65°C for 15 s; and 72°C for 30 s); and a final extension of 72°C for 5 min. The numbers of cycles were tested to ensure that they fell within the linear phase of amplification. Amplicons (~450 bp) of 4 reactions for each sample were pooled, size-selected and purified with Agencourt AMPure XP beads (Beckman Coulter, Inc.) at a 0.8 ratio. The second step of PCR was performed with two separate 50 µl reactions with 50 ng of first step purified PCR product per reaction. NEBNext Multiplex Oligos for Illumina (Dual Index Primers) were used to attach Illumina adapters and indices to the samples. The thermocycling parameters were: 95°C for 3 min; 6 cycles of (98°C for 20 s; 65°C for 15 s; 72°C for 30 s); and 72°C for 5 min. The amplicons from these two reactions for each sample were pooled, size-selected and purified with Agencourt AMPure XP beads at an 0.8 ratio. The purified second-step PCR library was quantified by Qubit dsDNA HS assay (Thermo Fisher Scientific) and used for downstream sequencing on an Illumina HiSeq platform.

For amplification of barcodes from genomic DNA, genomic DNA was extracted from stored cell pellets with a DNeasy Blood and Tissue Kit (Qiagen). The first step PCR was performed as two separate 50 µl reactions for each sample. 500 ng of genomic DNA was input per reaction with Kapa Hifi Hotstart ReadyMix. The PCR primers used were, sgRNAsequenceAmp_gDNA_F: ACACTCTTTCCCTACACGACGCTCTTCCGATCTTATATATCTTGTGGAAGGACGAAA

CACCG and sgRNAsequenceAmp_gDNA_R: GACTGGAGTTCAGACGTGTGCTCTTCCGATCTCCTTATTTTAACTTGCTATTTCTAGCTCTA. The thermocycling parameters were: 95°C for 3 min; 24–32 cycles of (98°C for 20 s; 60°C for 15 s; and 72°C for 30 s); and a final extension of 72°C for 5 min. The numbers of cycles were tested to ensure that they fell within the linear phase of amplification. Amplicons (~150 bp) of the two reactions for each sample were pooled, size-selected with Agencourt AMPure XP beads at a ratio of 1.6. The second step of PCR was performed as two separate 50 μl reactions with 25 ng of first step purified PCR product per reaction. NEBNext Multiplex Oligos for Illumina (Dual Index Primers) were used to attach Illumina adapters and indices to the samples. The thermocycling parameters were: 95°C for 3 min; 6–8 cycles of (98°C for 20 s; 65°C for 20 s; 72°C for 30 s); and 72°C for 2 min. The amplicons from these two reactions for each sample were pooled, size-selected with Agencourt AMPure XP beads at a ratio of 1.6. The purified second-step PCR library was quantified by Qubit dsDNA HS assay (Thermo Fisher Scientific) and used for downstream sequencing on an Illumina NovaSeq platform.

Single Nuclei Sequencing Library Generation

To extract nuclei, teratoma tissues frozen in OCT were processed as previously described (dx.doi.org/10.17504/protocols.io.5qpvoby69l4o/v2). Extracted nuclei were passed through a 50 μm filter, followed by a 30 μm filter and pelleted by spinning at 900 rcf at 4 ºC for 10 minutes. Resuspended nuclei in PBS + 1% BSA solution were stained for DAPI and viable nuclei were collected via FACS. Sorted nuclei were pelleted under the same condition and resuspended in PBS + RNase Inhibitors for generating single nucleus sequencing libraries using Chromium Single Cell 3' Reagent Kits v3 (10x Genomics).

Cell Type Annotation

Annotations of cell types were carried out by transferring previous annotations on the wild type teratoma using scANVI [4] and Seurat label transfer [5]. Cells with predictive scores greater than 0.8 were annotated and marker gene expression were checked for each annotated cell type and unannotated cell cluster.

Differentially Expressed Gene and Gene Ontology Analysis

The FindMarkers command in the Seurat R package was used to identify differentially expressed marker genes. Wilcoxon rank-sum test was used with other arguments set as the following: min.pct = 0.1, logfc.threshold = 0.25. At least 3 cells were required for comparison. Adjusted p-values were calculated using Benjamin-Hochberg procedure. All

differential genes that were statistically significant were inputted into the PANTHER classification system to identify enriched gene ontology terms [6].

iPSC to MSC Differentiation

KOLF2.1Js and PGP1s were differentiated into MSCs as previously described [7,8]. Briefly, hESCs were dissociated into EBs and then were plated on Matrigel coated plates in MSC differentiation medium (α-MEM + GlutaMAX (Gibco), 10% FBS (Gibco), 1% penicillin/streptomycin (Gibco), 10 ng/mL FGF2 (Peprotech) and 5 ng/mL TGFβ (Peprotech). About 10 days later, the confluent MSC-like cells were passaged on a Matrigel coated plate and cultured in MSC culture medium: 90% α-MEM + Glutamax (Gibco), 10% FBS (Gibco), 1% penicillin/streptomycin (Gibco) and 1 ng/mL FGF2 (Peprotech). Then CD73/CD90/CD105 antibodies (Miltenyi Biotec) were used to FACS sort for tripositive cells.

shRNA Lentiviral Production and Transduction of MSCs

shControl (Sigma Aldrich SHC002) and shADAR (Sigma Aldrich TRCN0000050788) plasmids were packaged into lenvirus as described above.

For viral transduction, on day −5, passage 4 MSCs were dissociated to a single cell suspension and seeded into 48 well plates at a density of 5,000 cells per well in MSCGM. On day -4 cells were then transduced with lentivirus added to fresh MSCGM containing polybrene (5 µg/ml). Media was replaced with fresh MSCGM on day -3, and then again on day -2 with MSCGM + Puromycin (10 µg/ml, Thermo Fisher Scientific). After 2 days of selection, media is replaced with Stempro Adipogenic Induction Media + Puromycin (10 µg/ml) to induce adipogenic fate, and media is replaced every 2-3 days.

Oil O Red and DAPI Staining

3.5 mg/mL Oil Red O Stock Solution (Sigma Aldrich O-0625) is made by dissolving Oil O Red in 100% isopropanol. Oil O Red working solution is made by mixing the Oil O red stock solution with ddH20 at 3:2 ratio. Oil O red working solution is set to sit at room temp for 20 min followed by filtering (0.2 µm).

Cells are fixed with 4% Paraformaldehyde for 1 hour at RT, washed 3x in ddH20, followed by applying Oil O red working solution for 10 minutes at RT. Cells are rinsed with ddH20 3x, followed by adding 1 µg/mL of DAPI solution (Thermo Fisher Scientific 62248). Cells are finally rinsed with ddH20 3x and subsequently imaged via brightfield and fluorescence microscopy.

Transcriptomic Maturity Correlation Between Fetal Brain and Teratoma Neural Tissue

To evaluate the developmental maturity of teratoma neural cell types, we first obtained the average expression levels of the neuro-ectoderm associated cells, including astrocytes, excitatory neurons and inhibitory neurons. We then calculated the cosine similarity between the average expression profiles of these cells to those of the neural cells in the human fetus at various developmental stages. Genes that are detected in both datasets and highly variable in either dataset were utilized.

RNA Editing Analysis in Cerebral Organoids

RNA sequencing data for cerebral organoids was retrieved via Synapse: syn26346373 [9]. AEI measurements were performed as mentioned above.

Teratoma Screen Guide Calling

To assign the guide identity to each cell in the teratoma screen, the guide count matrix was first obtained from CellRanger v3.0.1. The total number of guides for each cell was calculated and the guide identity for each cell was called for a cell with at least 5 guide counts by assigning the identity of the guide with the maximum number of guide counts expressed. For cells having more than 1 maximum guide count, the cells were annotated as 'multiplet' and removed from downstream analysis.

Germ-Layer and Pan-Teratoma Fitness Analysis

For germ-layer fitness analysis, the pre-injection frequency values for each unique guide were quantified from pre-injection ADAR-KO cell gDNA counts. After teratoma harvest, cell counts for each unique guide were added up for each germ layer, and then divided by the total number of cells within that germ layer. This frequency (post-harvest frequency) was divided by the pre-injection guide frequency (pre-injection frequency) of each unique guide, and then transformed with log2-scaling, resulting in the log2(Fold-Change) value. Within each teratoma, log2(Fold-Change) values for unique guides targeting the same gene were averaged, and n=4 log2(Fold-Change) values were plotted using 4 unique ADAR-KO teratomas.

Cell counts from Adipogenic MSCs, Chondrogenic MSCs, Cycling MSCs, Hematopoietic Cells, Kidney Progenitors, MSC-Fibroblasts, Muscle, Myo-Fibroblasts, Pericytes, and Smooth Muscle Cells were summed to calculate the total number of mesodermal cells.

Cell counts from Neural-Progenitors, Neurons, Retinal-Epithelium, and Schwann Cell Progenitors were summed to calculate the total number of ectodermal cells.

Cell counts from the Gut-Epithelium and Pancreatic Progenitors were summed to calculate the total number of endodermal cells.

For pan-teratoma fitness analysis, the total number of cells for each unique guide were added up, and then divided by the total number of cells in the teratoma. Subsequent calculations were conducted as described above.

WGCNA Gene Set Analysis

To contrast the effects on the transcriptome in the knock-out screen, co-expressed genes for each cell type were identified and the gene expression were gauged for each perturbation using the WGCNA gene set testing pipeline [10]. First, gene modules of the co-expressed genes within each cell type were identified using WGCNA R package [11]. We then calculated the gene-set score for each WGCNA module and used the R function lm to construct a linear regression model for examining how the gene-set scores vary in each perturbation. Teratoma batch differences and sequencing depths variances were corrected. FDR corrected p-values and effect sizes from the linear regression test were calculated.

Teratoma Cell-Type Enrichment Analysis

To compare the cell type composition in the knock-out screen, we adopted a previously established pipeline [10]. Specifically, a Poisson regression model was used to measure the effect size of cell type composition changes with corrected experimental batch differences and extracted p-values were corrected using Benjamin-Hochberg procedure.

SUPPLEMENTARY REFERENCES

1. McDonald, D. *et al.* Defining the Teratoma as a Model for Multi-lineage Human Development. *Cell* 183, 1402–1419.e18 (2020).

2. Doench, J. G. *et al.* Optimized sgRNA design to maximize activity and minimize off-target effects of CRISPR-Cas9. *Nat. Biotechnol.* 34, 184–191 (2016).

3. Zheng, G. X. Y. *et al.* Massively parallel digital transcriptional profiling of single cells. *Nat. Commun.* 8, 14049 (2017).

4. Xu, C. *et al.* Probabilistic harmonization and annotation of single-cell transcriptomics data with deep generative models. *Mol. Syst. Biol.* 17, e9620 (2021).

5. Stuart, T. *et al.* Comprehensive Integration of Single-Cell Data. *Cell* 177, 1888–1902.e21 (2019).

6. Thomas, P. D. *et al.* PANTHER: Making genome-scale phylogenetics accessible to all. *Protein Sci.* 31, 8–22 (2022).

7. Jakob, M. *et al.* Pluripotent Stem Cell-Derived Mesenchymal Stem Cells Show Comparable Functionality to Their Autologous Origin. *Cells* 10, (2020).

8. Wu, Z. *et al.* Differential stem cell aging kinetics in Hutchinson-Gilford progeria syndrome and Werner syndrome. *Protein Cell* 9, 333–350 (2018).

9. Trujillo, C. A. *et al.* Complex Oscillatory Waves Emerging from Cortical Organoids Model Early Human Brain Network Development. *Cell Stem Cell* 25, 558–569.e7 (2019).

10. Jin, X. *et al.* In vivo Perturb-Seq reveals neuronal and glial abnormalities associated with autism risk genes. *Science* 370, (2020).

11. Langfelder, P. & Horvath, S. WGCNA: an R package for weighted correlation network analysis. *BMC Bioinformatics* 9, 559 (2008).
