# Supplementary material for: Charting and probing the activity of ADARs in human development and cell-fate specification
Source: Nat Commun. 2024 Nov 13;15:9818. doi: 10.1038/s41467-024-53973-0 (PMC11561244; doi:10.1038/s41467-024-53973-0)
Supplement: Supplementary file 2 — Reporting Summary [file 41467_2024_53973_MOESM2_ESM.pdf]

nature portfolio

Corresponding author(s): Prashant Mali

Last updated by author(s): 10/4/2024

Reporting Summary

Nature Portfolio wishes to improve the reproducibility of the work that we publish. This form provides structure for consistency and transparency in reporting. For further information on Nature Portfolio policies, see our Editorial Policies and the Editorial Policy Checklist.

Statistics

For all statistical analyses, confirm that the following items are present in the figure legend, table legend, main text, or Methods section.

n/a	Confirmed	
[ ]	[x]	The exact sample size (*n*) for each experimental group/condition, given as a discrete number and unit of measurement
[ ]	[x]	A statement on whether measurements were taken from distinct samples or whether the same sample was measured repeatedly
[ ]	[x]	The statistical test(s) used AND whether they are one- or two-sided<br>*Only common tests should be described solely by name; describe more complex techniques in the Methods section.*
[x]	[ ]	A description of all covariates tested
[x]	[ ]	A description of any assumptions or corrections, such as tests of normality and adjustment for multiple comparisons
[x]	[ ]	A full description of the statistical parameters including central tendency (e.g. means) or other basic estimates (e.g. regression coefficient) AND variation (e.g. standard deviation) or associated estimates of uncertainty (e.g. confidence intervals)
[ ]	[x]	For null hypothesis testing, the test statistic (e.g. *F*, *t*, *r*) with confidence intervals, effect sizes, degrees of freedom and *P* value noted<br>*Give P values as exact values whenever suitable.*
[x]	[ ]	For Bayesian analysis, information on the choice of priors and Markov chain Monte Carlo settings
[x]	[ ]	For hierarchical and complex designs, identification of the appropriate level for tests and full reporting of outcomes
[ ]	[x]	Estimates of effect sizes (e.g. Cohen's *d*, Pearson's *r*), indicating how they were calculated

Our web collection on statistics for biologists contains articles on many of the points above.

Software and code

Policy information about availability of computer code

Data collection	RNA sequencing data for teratomas was retrieved via NCBI GEO accession code GSE: 156170.<br>RNA sequencing data for fetal organs was retrieved via raw data provided at dbGaP (accession number phs002003.v1.p1).<br>RNA sequencing data for cerebral organoids was retrieved via Synapse: syn26346373.<br>RNA sequencing data for all bulk human organs was retrieved on Array Express via accession code E-MTAB-6814.
Data analysis	Alu editing Index (AEI) was calculated using the following github: https://github.com/a2iEditing/RNAEditingIndexer<br>Site-Specific Editing Analysis was done using REDITools from the following github: https://github.com/BioinfoUNIBA/REDItools<br>GraphPad prism version 9.0.0 was used for plotting figures and computing associated p values<br>FIJI ImageJ was used for Oil O Red image Cell Percentage Analysis.

For manuscripts utilizing custom algorithms or software that are central to the research but not yet described in published literature, software must be made available to editors and reviewers. We strongly encourage code deposition in a community repository (e.g. GitHub). See the Nature Portfolio guidelines for submitting code & software for further information.

Data

Policy information about availability of data

All manuscripts must include a data availability statement. This statement should provide the following information, where applicable:

- Accession codes, unique identifiers, or web links for publicly available datasets
- A description of any restrictions on data availability
- For clinical datasets or third party data, please ensure that the statement adheres to our policy

Source data are provided with this paper. All scRNA-seq data will be made available via GEO.

Research involving human participants, their data, or biological material

Policy information about studies with human participants or human data. See also policy information about sex, gender (identity/presentation), and sexual orientation and race, ethnicity and racism.

Reporting on sex and gender	n/a
Reporting on race, ethnicity, or other socially relevant groupings	n/a
Population characteristics	n/a
Recruitment	n/a
Ethics oversight	n/a

Note that full information on the approval of the study protocol must also be provided in the manuscript.

Field-specific reporting

Please select the one below that is the best fit for your research. If you are not sure, read the appropriate sections before making your selection.

- [x] Life sciences
- [ ] Behavioural & social sciences
- [ ] Ecological, evolutionary & environmental sciences

For a reference copy of the document with all sections, see nature.com/documents/nr-reporting-summary-flat.pdf

Life sciences study design

All studies must disclose on these points even when the disclosure is negative.

Sample size	At least 2 independent samples per tested condition were evaluated for most experiments. No statistical methods were used to predetermine sample size.
Data exclusions	No data was excluded.
Replication	2 or more independent biological replicates were evaluated for both in vitro studies in cultures cells and in vivo studies using mice. Findings were consistent in all replicates.
Randomization	n/a
Blinding	n/a

Reporting for specific materials, systems and methods

We require information from authors about some types of materials, experimental systems and methods used in many studies. Here, indicate whether each material, system or method listed is relevant to your study. If you are not sure if a list item applies to your research, read the appropriate section before selecting a response.

Materials & experimental systems

n/a	Involved in the study	
	x	Antibodies
	x	Eukaryotic cell lines
x		Palaeontology and archaeology
	x	Animals and other organisms
x		Clinical data
x		Dual use research of concern
x		Plants

Methods

n/a	Involved in the study	
x		ChIP-seq
	x	Flow cytometry
x		MRI-based neuroimaging

Antibodies

Antibodies used	130-124-011 CD73-APC, human, AD2, 30 tests<br>130-114-860 CD90-PE, human, REA897, 100 tests<br>130-098-778 CD105-FITC, human 30 tests
Validation	Validated by the vendors.

Eukaryotic cell lines

Policy information about cell lines and Sex and Gender in Research

Cell line source(s)	H1, H9, HUES62, KOLF2.1Js, and PGP1 PSC cell lines were utilized in this study.
Authentication	n/a
Mycoplasma contamination	These cell lines were all tested for mycoplasma contamination in house. No contamination was found.
Commonly misidentified lines<br>(See ICLAC register)	n/a

Animals and other research organisms

Policy information about studies involving animals; ARRIVE guidelines recommended for reporting animal research, and Sex and Gender in Research

Laboratory animals	NOD.Cg-Prkdc scid Il2rg tm1Wjl /SzJ|, 5-10 week old male mice were used to grow teratomas.
Wild animals	Study did not involve wild animals.
Reporting on sex	5-10 week old male mice were used to grow teratomas. We do not expect the sex of the mouse to be relevent to teratoma development, and male mice were solely used to remain consistent across experiments.
Field-collected samples	Study did not involve samples collected from the field.
Ethics oversight	Institutional Animal Care and Use Committee of the University of California, San Diego.

Note that full information on the approval of the study protocol must also be provided in the manuscript.

Plants

Seed stocks	n/a
Novel plant genotypes	n/a
Authentication	n/a

Flow Cytometry

Plots

Confirm that:

☐ The axis labels state the marker and fluorochrome used (e.g. CD4-FITC).

☐ The axis scales are clearly visible. Include numbers along axes only for bottom left plot of group (a 'group' is an analysis of identical markers).

☐ All plots are contour plots with outliers or pseudocolor plots.

☐ A numerical value for number of cells or percentage (with statistics) is provided.

Methodology

Sample preparation	H1 and iMSC cells were cultured and dissociated using standard procedures.
Instrument	BD FACSAria II Cell Sorter
Software	FlowJo
Cell population abundance	At least 10k gated cells were analysed per sample.
Gating strategy	Gates were drawn to exclude cell debris and clumps. H1 cells served as a negative control for CD73 and CD105 sorting

☐ Tick this box to confirm that a figure exemplifying the gating strategy is provided in the Supplementary Information.
